# Pathological Findings in Two Events of Illegal Hunting of Atlantic Cory’s Shearwater (*Calonectris borealis*) from the Canary Islands

**DOI:** 10.3390/ani16081280

**Published:** 2026-04-21

**Authors:** José Navarro-Sarmiento, Ayoze Castro-Alonso, Gustavo Montero-Hernández, Lucía Marrero-Ponce, Antonio Fernández, Cristian M. Suárez-Santana

**Affiliations:** Unit of Veterinary Histology and Pathology, University Institute of Animal Health and Food Safety (IUSA), Veterinary School, University of Las Palmas de Gran Canaria (ULPGC), 35413 Las Palmas de Gran Canaria, Canary Islands, Spain; jose.navarro107@alu.ulpgc.es (J.N.-S.); gustavo.hernandez@ulpgc.es (G.M.-H.); lucia.marrero@ulpgc.es (L.M.-P.); antonio.fernandez@ulpgc.es (A.F.); cristian.suarez@ulpgc.es (C.M.S.-S.)

**Keywords:** Atlantic Cory’s shearwater, illegal hunting, forensic pathology, cranioencephalic trauma, Canary Islands, radiological imaging

## Abstract

This study characterizes the pathological findings in Atlantic Cory’s shearwater (*Calonectris borealis*) chicks from the Canary Islands associated with illegal hunting cases. A retrospective analysis of necropsy archives, including imaging techniques (X-rays and computed tomography) and histopathological examinations, was conducted on twenty juvenile shearwaters. The study revealed significant cranioencephalic trauma as the primary cause of death in both poaching events. Differences in the methods used by the perpetrators were noted, with variations in skull fractures and associated injuries. These findings highlight the importance of understanding local hunting techniques and sociocultural factors in forensic investigations. Diagnostic imaging, standardized necropsy, and histopathology are essential tools for the forensic investigation of illegal hunting in veterinary sciences.

## Simple Summary

This research examines the deaths of Atlantic Cory’s shearwater chicks in the Canary Islands associated with illegal hunting. By analyzing necropsy records, X-rays, computed tomography, and tissue samples, the study found that head injuries were the main cause of death; differences were noted by hunting methods. Understanding local hunting practices and cultural factors is crucial for forensic investigations. The study highlights the importance of diagnostic imaging, necropsy, and histopathological analysis in the investigation of illegal hunting cases.

## 1. Introduction

The Atlantic Cory’s shearwater (*Calonectris borealis*) belongs to the family *Procellariidae*. Like other procellarids, the chicks leave their natal colony. Then, after several years at sea, they return to land for their first breeding cycle. In the Macaronesian population, individuals return to land between February and March, with egg-laying occurring from late May to early June. Following an incubation period of approximately 54 days, hatching takes place in July, and chicks fledge between late October and early November [1]. These birds nest on cliffs near the coast, often in areas of difficult access, on rocks, or in burrows [2].

The hunting of chicks by hand has been documented throughout the Canary Islands archipelago, particularly in the eastern region (Fuerteventura, Lanzarote, La Graciosa and the small islets of the Chinijo archipelago) [3,4,5]. Poaching has reduced the breeding success of the affected colonies by almost a third. Considering their breeding and ecological behavior, it is estimated that illegal hunting could play a main role in the species’ extinction within 20–40 years [4].

Although the International Union for the Conservation of Nature (IUCN) classifies the Atlantic Cory’s shearwater as a least concern [6], certain populations, such as those in the Mediterranean and Macaronesian regions, are considered vulnerable [7]—mainly due to pressures such as illegal hunting, accidental capture and light pollution [8,9,10]. In addition, this bird is listed in Spain under the List of Wild Species under Special Protection Regime (LESPRE). As a result, national and international laws prohibit their capture or commercialization [11,12].

In this article, we describe the postmortem findings of several Cory’s shearwater chicks from two different cases of illegal hunting that were judicially processed. Using a forensic pathology approach, we aim to answer the questions: How and why did these animals die?

## 2. Materials and Methods

### 2.1. Study Design

This study is presented in the context of veterinary forensic pathology. The circumstances of death and the necropsy results are described to try to determine the cause of death, mechanism of death, and manner of death [13].

We retrospectively analyze necropsy archives comprising over 3800 wild birds found deceased in the Canary Islands and submitted for necropsy. This is part of a broader survey and monitoring initiative on the health of the Canarian wildlife, conducted by the Canarian Government in accordance with European Regulations (European Regional Development Fund), under the coordination of the Red Vigía Canarias [14]. The eligibility criteria required: (1) that trauma was identified as the primary cause of death; and (2) that the death was attributable to poaching, based on the autopsy findings and contextual forensic evidence. Specimens were excluded if they belonged to avian species other than Cory’s shearwater, if the cause of death was not consistent with traumatic injury, or if the manner of death was due to natural causes or accidental events. A total of twenty specimens of Cory’s shearwater were included in the study. All the individuals included in this article were involved in two episodes of illegal hunting that were judicially processed and resulted in the condemnation of the perpetrators. Ethical review and approval were waived for this study since the specimens included were found dead and no animals were manipulated alive or euthanized.

### 2.2. Circumstances of Death

**Figure 1:**
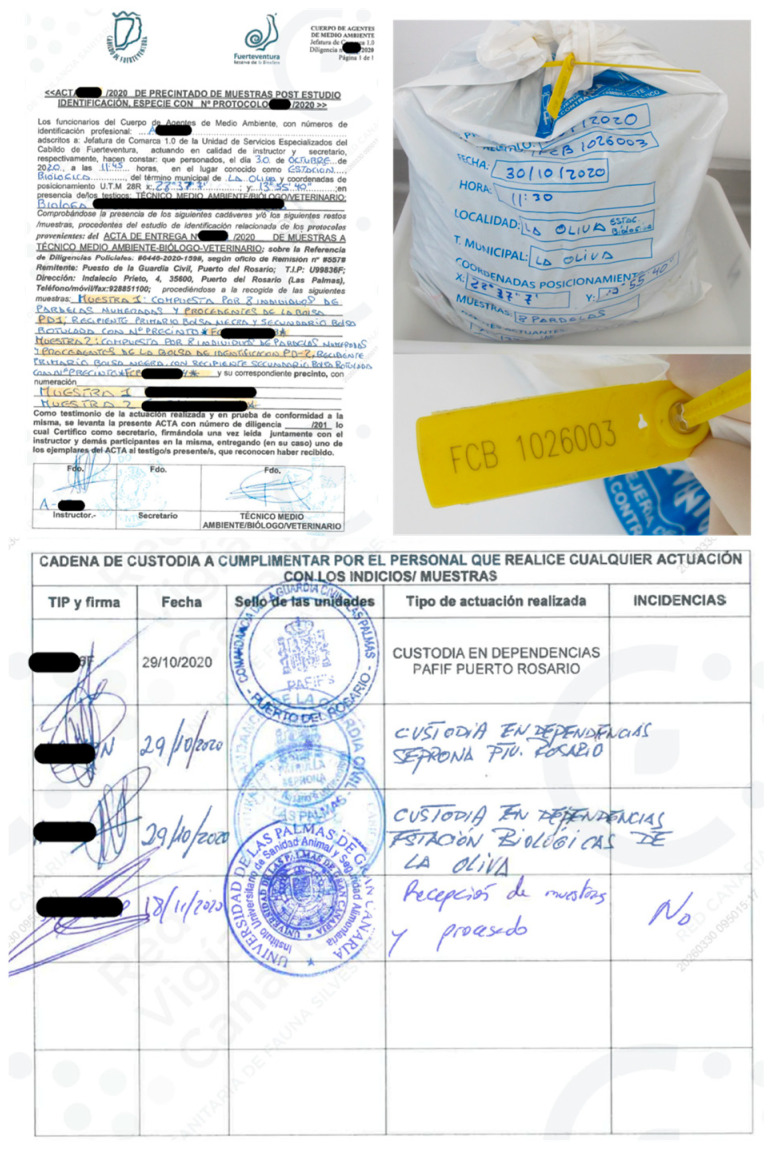
Official report that accompanied the corpses (**top left**). The samples were submitted in sealed plastic bags, individually identified with a numbered plastic seal (**top right**), together with the respective chain of custody (**bottom**).

The bodies were collected by local authorities with specific competences in crimes against the fauna (the Nature Protection Service of the Civil Police and the Environmental Police). Police officers were responsible for the recovery of the carcasses, the crime scene inspection, the collection of evidence, and the initiation of the chain of custody. They also identified the suspects involved in the alleged wildlife crimes and initiated the corresponding legal complaint to trigger judicial investigation. After the recovery of the carcasses and the collection of evidence, the officers transferred the seized animals for forensic necropsies and the determination of the causes of death. The carcasses were delivered inside sealed plastic bags, each one labeled with the location, time, and date of recovery; the species; the case reference number; and an official report detailing all documented circumstances surrounding the deaths. Figure 1 illustrates the official report that accompanied the samples (corpses), how the samples were identified, and the chain-of-custody (note that all the sensitive information has been censured for confidentiality).

The information regarding the circumstances of death was compiled from the reports accompanying the sample submissions and the chain of custody of the samples. These included the exact location of the bodies, the dates, the case references, and descriptions of the scenes. They were written by the police authorities involved in the cases. In one case, the documents questioned the possibility that a ferret might have interacted with the birds before their death. In the other case, the police confiscated the corpses from suspected hunters and requested a full necropsy report.

### 2.3. Necropsy

After the reception and verification of the identity of the samples, X-ray examinations (50 kV, 10 mA) were performed prior to the necropsies for all cases, with the carcasses kept inside the bags during the imaging process. In most cases, four radiographic projections were performed: two whole-body oblique views, one cephalic in the same orientation, and one lateral projection of the head and neck. Additionally, computed tomography (CT) scans were conducted on six animals (cases Nº 2, 3, 6, 7, 8, and 11) using a Helical CT scanner (Toshiba Astelion, Canon Medical System^®^, Tokyo, Japan). The protocol was adjusted for animals weighing less than 5 kg, with settings of 120 kVp and 80 mA. Serial cross-sectional images were obtained with a thickness of 1 mm and overlapping slices. For image visualization and reconstruction, specific software (RadiAnt DICOM Viewer 2025.1, Medixant, Poznan, Poland) was utilized.

A complete standardized necropsy was performed on each individual, following a modification of previously published protocols [15,16,17]. Briefly, after documenting the external container and verifying the identity of the sample and the chain of custody, an external examination of the carcass and body mass was conducted. Feathers were carefully removed, and the skin was inspected and dissected to allow visualization of the subcutaneous tissue. Nutritional condition was assessed using pectoral muscle mass and subcutaneous and visceral fat. It was classified on a scale of 1 to 3 (1: cachexia; 2: thinness; 3: normal), modifying the proposal of Burton et al. (2014) [18]. Body cavities were opened for in situ visceral inspection. All viscera were removed from the body, individually inspected, and sampled for histopathology. The musculoskeletal system was thoroughly inspected, including the opening of joints and inspection of the vertebral column, synsacrum, and cranium. Finally, in all the animals the cranial bones were carefully removed to expose the dura mater and encephalon.

Samples for histopathology included the adrenal gland, air sacs, bursa of Fabricius, cloaca, encephalon, eyes, feathers, gizzard, gonads, heart, intestine, kidney, larynx, liver, lungs, pancreas, pectoral muscle, proventriculus, skin, spleen, thymus, tongue, trachea, and ureter. Tissue samples were fixed in 10% neutral buffered formalin for 24 h and routinely processed for histological analysis. The samples were dehydrated through several passes in increasingly graded alcohol and xylene before paraffin embedding. Sections of 5 micrometers were obtained and stained with hematoxylin and eosin for histopathological examination. Additionally, Masson Trichrome and Giemsa stains were performed to better characterize certain histological findings.

### 2.4. Statistical Analysis

The comparison of the proportion between the two events of illegal hunting was performed by Pearson’s Chi-squared test. Significant differences were considered when α ≤ 0.05. Statistical analysis was performed using Excel (Microsoft v.2016) and Python (v3.11).

## 3. Results

The 20 birds were all categorized as juveniles (chicks), based on the degree of skeletal ossification, the presence of down, and juvenile feathers in the primary and secondary remiges. The median animal weight was 741.2 ± 179.1 g. 70% of the animals were male (*n* = 14), while 30% were female (*n* = 6). According to the origin of the carcasses, 16 individuals came from Fuerteventura, whereas four came from Lanzarote. The bodies were found between late September and October. All the carcasses were frozen for conservation until submission for postmortem analysis. The carcasses were kept frozen (−20°) for 2–3 weeks and transported inside coolers. The samples were kept in refrigeration (4 °C) for 24 h before necropsy. Detailed information about the cases can be found in Table 1.

### 3.1. Circumstances of the Death

**Figure 2:**
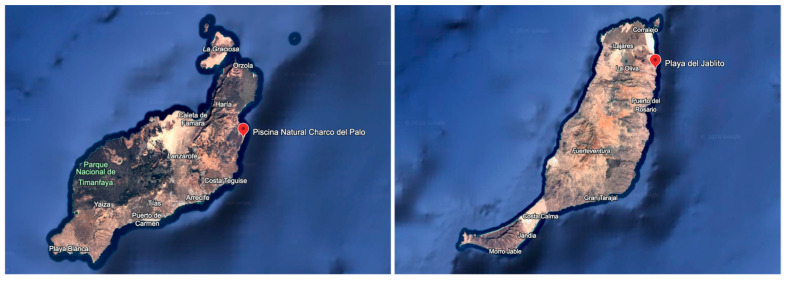
Distribution of the first episode in Lanzarote (**left**) and the second episode in Fuerteventura (**right**). Images obtained from Google Earth © [2026] Google, Maxar Technologies [19].

All the bodies were submitted for necropsy by the local authorities in sealed and labeled plastic bags. The exact locations of the events are not disclosed due to confidentiality. However, the carcasses correspond to two different episodes: one in Lanzarote (Episode 1) and the other in Fuerteventura (Episode 2). The approximate locations are depicted in Figure 2.

In both events, the police apprehended furtive hunters in possession of Cory’s shearwater corpses. In the first episode, the police specifically requested an evaluation to determine if the wounds observed on the birds could have been inflicted by a ferret (*Mustela putorius furo*), as the offenders were using ferrets for hunting. The individuals involved, locations, and dates of the episodes were initially unrelated.

### 3.2. Radiological Imaging

**Figure 3:**
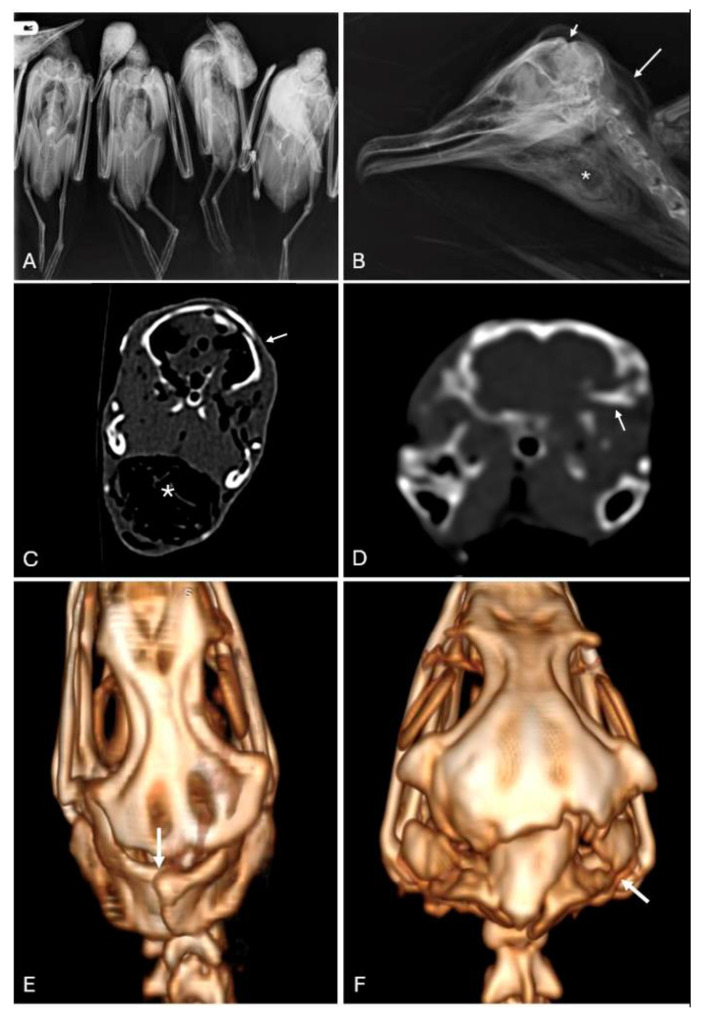
Radiological images of the two episodes including X-rays (**A**,**B**), transverse CT section of the head (**C**,**D**), and 3D volume rendering (**E**,**F**). (**A**) Cases 5–8. Absence of ballistic material or long bone fractures. (**B**) Case 1. Fracture of the neurocranium (short arrow) associated with focal subcutaneous emphysema (long arrow). The pharynx is markedly enlarged by foreign material (asterisk). (**C**) Case 2. Lateral collapse of the neurocranium associated with overlapping of the sutura interpariatelis (arrow). The pharynx is enlarged by air and foreign material (asterisk). (**D**) Case 6. Bilateral fractures of the parietal bone, with concurrent basioccipital fracture (arrow). (**E**) Case 2, 3D reconstruction. Lateral collapse of the neurocranium associated with overlapping of the sutura interpariatelis (arrow). (**F**) Case 6, 3D reconstruction. Bilateral fractures of the parietal bone, with concurrent basioccipital fracture (arrow).

Whole-body X-rays examinations confirmed the absence of projectiles in the bodies (Figure 3A). No fractures involving long bones were observed in any of the individuals. In four cases (Episode 2), skull fractures were noted on the radiographic studies. These included fractures of the neurocranium and subcutaneous emphysema in the head and neck subcutis. Additionally, expansion of the pharynx by radiologically heterogeneous (soft tissue and air) radiodensity foreign material was noted in the four individuals involved in the Lanzarote event (Figure 3B).

Regarding CT analysis, the main pathological findings were focused on the skull. The neurocranium of all six analyzed individuals showed fractures involving the cranial sutures (Figure 3C) or bones, associated with displacement of the neurocranium and collapsing of the calvaria (Figure 3D). The CT scans revealed less ossification of the cranial bones in the two cases from the first episode (Figure 3E) compared to the cases from the second episode (Figure 3F). These individuals still presented non-ossified cranial sutures. The sutura interparietalis and the sutura frontoparietalis were overlapping, causing intrusion of the parietal bones.

### 3.3. Gross Pathology

**Figure 4:**
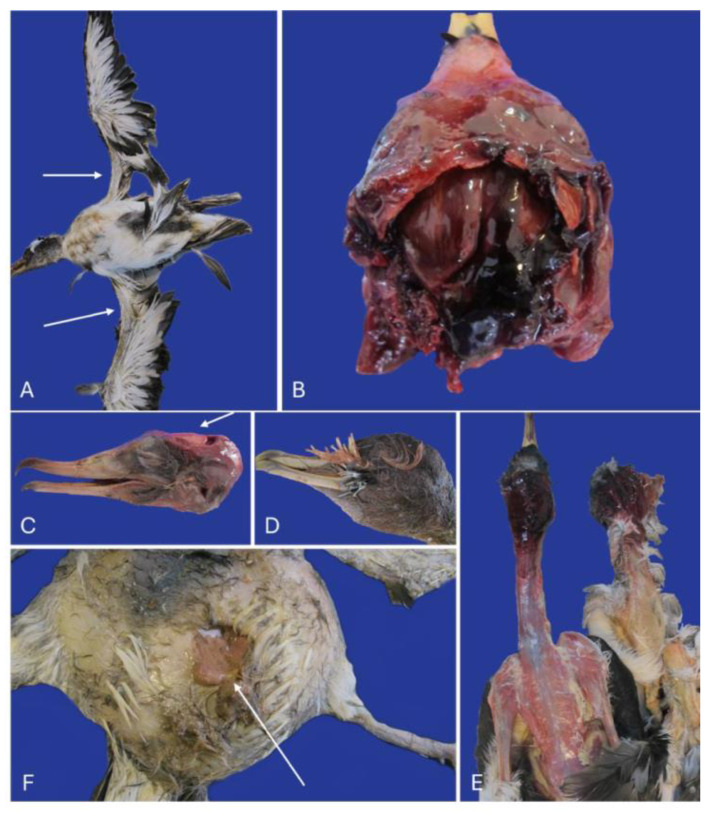
Gross pathology. (**A**) Case 2. Absence of multiple secondary remiges and secondary coverts(arrows). (**B**) Case 17. Extensive subdural hematoma associated with cranial bone fracture. (**C**) Case 2. Deformation of the neurocranium (arrow). (**D**) Case 3. Presence of feathers in the oral cavity. (**E**) Case 9. Extensive subcutaneous hemorrhage (**F**) Case 1. Celomic perforation (arrow).

Externally, all the animals from the second event maintained the integrity of their plumage, whereas in the four cases from the first event, multifocal areas of alopecia were found on the trunk and wings (Figure 4A). Profuse epistaxis was noted in 71% of the animals (*n* = 10) from the second event. The epistaxis was observed originating from the naris or nasopharynx.

The most consistent finding observed in all the animals from both events was the fracture of the cranium with extensive cranioencephalic hemorrhage (Figure 4B). The fracture frequently caused central intrusion of the neurocranium and displacement of the calvaria (Figure 4C), with concurrent fractures of the os parietale and os basioccipitale. In other cases, the fracture involved the os supraoccipitale or the rostrum parasphenoidale. In some individuals, cranial deformation was notable upon external examination (Figure 3D). The cranial hemorrhages extended to the cervicocephalic subcutaneous tissue, causing blood infiltration through the cranial and facial skin (Figure 4E).

In the four individuals from Lanzarote, there was a characteristic symmetrical feather loss of the primary remiges in the medial region of the wings. The missing feathers were found obstructing the oropharynx in all four cases from this event. Other areas of alopecia coincided with incised wounds affecting the skin. The carcasses showed abnormal positions, including abducted or extended wings and disorganized feathers, interpreted as signs of fighting. Multifocal incised wounds were noted in the four animals in various body regions, including the cervical, cranial, and trunk areas. Most injuries were linear incised wounds affecting both the skin and subcutaneous tissue, approximately 1 cm in length, often accompanied by subcutaneous hemorrhages. In one case, the injury extended deeper, resulting in celomic perforation (Figure 4F).

**Figure 5:**
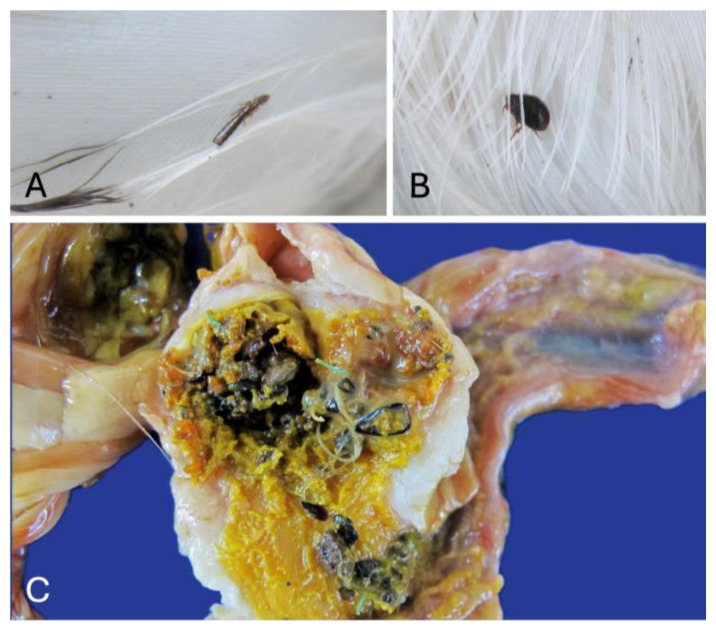
Gross pathology. (**A**,**B**) Case 2. Detail of ectoparasites observed on the feathers. (**C**) Case 13. Abundant presence of microplastics in the gizzard.

No fractures affecting long bones were detected, and all the carcasses showed abundant fat deposits in the subcutaneous tissue and coelomic cavity. The skeletal muscle was pale in all cases, interpreted as normal due to the young age of the individuals. Ectoparasites were noted in 75% of the cases (*n* = 15) (Figure 5A,B). As an incidental finding, small fragments of plastic (less than 1 mm) were frequently noted and registered as “relatively abundant” in 25% of the individuals (*n* = 5) (Figure 5C).

### 3.4. Histopathology

**Figure 6:**
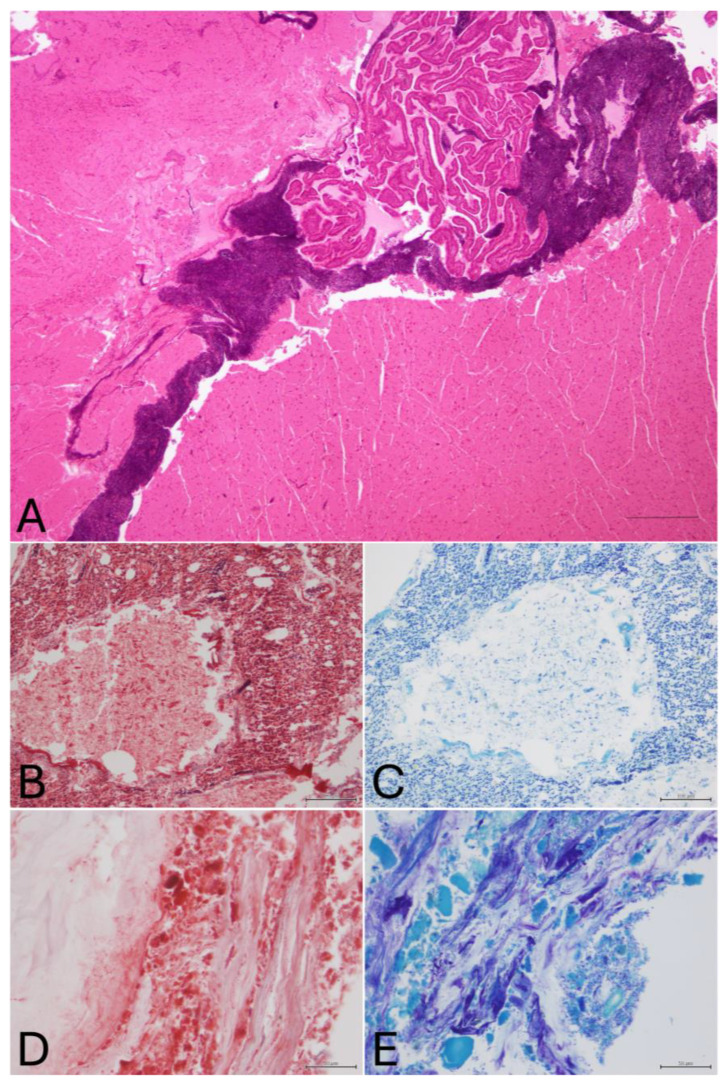
Histopathological findings. (**A**) Case 12. Severe hemorrhage in the lateral ventricle of the encephalon (HE, Scale 100 µm). (**B**,**C**) Case 3. Severe distention of the parabronchi (MT, Scale 100 µm), (Giemsa 20×) (**D**,**E**) Case 3. Foreign material collected from the pharynx (feathers), (MT and Giemsa, Scale 50 µm).

Histologically, the most relevant lesions were observed in the encephalon, consisting of hemorrhage filling the lateral ventricles (Figure 6A) and subarachnoid spaces. Multifocally, the neuroparenchyma was disrupted, and multiple perivascular and intraparenchymatous hemorrhages were noted. Encephalic hemorrhages were reported in all necropsied animals (*n* = 20). The nervous tissue in all the animals showed abundant freeze–thaw-related artifacts, visible as linear optically empty spaces frequently disrupting the parenchyma, lysis of erythrocytes, and poor cellular definition [20,21].

Hemorrhages in the parabronchi were noted in 15% of the cases (*n* = 3). Furthermore, in four cases (cases 1–4), foreign material was observed in the lumen of the parabronchi, consisting of amphophilic spicules. They were approximately 10 micrometers in diameter and of variable length, occasionally pigmented (Figure 6B,C). The content of the pharynx of these animals (feathers) was processed for histopathology and compared with the content of the parabronchi (Figure 6D,E). Both materials were similar in morphology and staining characteristics, revealing slight metachromasia in Giemsa and an abundant presence of numerous bacteria.

Additionally, multifocal hemorrhages were noted in the myocardium of 45% of the animals (*n* = 9), frequently located at the subendocardial level and in the papillary muscles of the left ventricle.

Renal trematodiasis was observed in 50% of cases (*n* = 10), with abundant embryonated trematode eggs with thick acidophilic cuticles being observed in the lumen of the collecting tubules.

### 3.5. Statistical Analysis

The different necropsy findings were organized by episodes and represented in Table 2. The chi-square analysis revealed that most findings did not differ significantly between episodes. Conditions such as skull fractures, cranial hemorrhages, bloodstained plumage, skeletal muscle necrosis, renal trematodiasis, cardiac hemorrhages, microplastic ingestion, and pulmonary hemorrhages showed no statistical association with the episode. In contrast, three lesions were found exclusively in Episode 1 and demonstrated highly significant differences. These were feathers in the oral cavity, incised skin wounds, and myocardial degeneration (*p* = 0.00016).

## 4. Discussion

We described the circumstances of death and the necropsy results of twenty illegally hunted chicks of Cory’s shearwaters in two different episodes. The cause of the death in all the animals was cranioencephalic trauma. However, differences were detected in the two episodes that indicate variations in the methods used by the perpetrators to capture and kill the animals in these two poaching events. There were lesions and findings that were observed only in Event 1, such as the presence of feathers in the oral cavity, incised wounds and myocardial degeneration.

The circumstances of death, in which the animals were seized by the police directly from the perpetrators, point out that the manner of death of these chicks was non-natural (violent/anthropic). Traumatic injuries in the cranial and cervical region were confirmed in both events of hunting. Moreover, the police suspected that ferrets were used to catch the birds in the first episode, which we consider to be important information regarding the circumstances of death. The presence of incised wounds, multiple subcutaneous hematomas, and patchy alopecia in the carcasses of that event supported the implication of small carnivores, which is in accordance with the officials’ suspicions.

The CT images illustrated in this work are comparable to those reported for turkey hens killed by percussive bolt shooting, which showed depressed cranial bones with overlapping fragments [22]. The individuals were osteologically immature, which could have facilitated manual fracture of the neurocranium. It has been reported that there is a higher incidence of skull trauma using non-penetrating captive bolt devices in young animals compared with adults in poultry species [23]. The avian skull is characterized by a high degree of fusion (synostosis) of its separate elements that occurs early in life; most often, the ankylosis is so complete that traces of the sutures may not be observed in mature individuals [24]. In our observations, we noted less ossification in the skulls from the first episode, indicating that younger individuals were involved in this poaching event.

All the carcasses were frozen before necropsy, which greatly affected the preservation of the nervous system. Despite that, histopathologic results confirmed the encephalic extension of the trauma, with findings similar to those reported during culling in poultry, which included massive subdural hematomas [25]. Based on the extent and location of the hemorrhages, potential vessels affected would include the ascending vertebral artery, cerebral carotid artery, cerebral arteries, occipital sinus, internal occipital vein, and cervical sinus, among others [26]. We could not identify cortical brain damage in the form of neuronal necrosis. This could be due to the lack of fine cellular detail secondary to the defrosting of the carcasses. Although freeze–thaw artifacts may obscure some lesions, particularly acute degenerative lesions, they do not preclude a sample from being histopathologically analyzed, especially in a forensic context [20,21,27].

Regarding the mechanism of death, in all the animals the fatal cranial trauma would have caused severe acute neural disfunction, with loss of consciousness, and cardiocirculatory and ventilatory insufficiency that would have rapidly resulted in death. However, in the first event, the external aspect of the animals indicated more self-defense before dying, with patchy alopecia coinciding with incised wounds, and the hunters were suspected of using ferrets to capture the birds. The fight against a carnivore, with severe incising and perforating injuries, could trigger an extreme catecholamine discharge that may contribute to a maintained tachycardia, which can be lethal by itself in a young chick. Moreover, after capture, suffocation could have contributed importantly to the mechanism of death in these four animals, as feathers were found in the pharynx, blocking the airway [28,29]. In one of them, traces of inhaled feathers were noted in the parabronchi, indicating that the animal was still breathing when the feathers were introduced into the pharynx.

It is of interest for veterinarians involved in forensic cases of illegal hunting to understand prey habits, the hunting techniques used, and the sociocultural implications of hunting in specific areas. Historically, the “harvesting” of seabirds on the Atlantic islands of Macaronesia, including the Azores, Madeira, and the Canary Islands, has been linked to population declines of species such as Cory’s shearwater. In the context of the Canary Islands, the hunting of Cory’s shearwater has been well-documented and intrinsically linked to the local population, even from precolonial times [4]. Popular hunting techniques for chicks involve hunters descending into cliffs to harvest the birds by hand or using a tie; however, the use of dogs, ferrets, and smoke has also been documented [4].

Regarding the use of the chicks, they have been considered part of the culinary culture in some specific areas of the eastern part of the archipelago. When a bird is captured for consumption, hunters typically avoid damaging the body or causing unnecessary hematomas that could depreciate the appearance of the meat. This may explain why, in the second event, the only trauma was located at the cranioencephalic level, while the rest of the carcasses were intact. However, to correctly interpret the findings observed in our first event, it is important to know that the oils extracted from the chick’s proventriculus are popularly reputed as medicinal [30]. This reputation can be sufficient in the black market for the oil to be the sole purpose of the hunting. We theorize that the introduction of the birds’ own feathers into the pharynx of the four individuals from the first event aimed to filter the stomach contents to extract cleaner oil once the animal was unconscious or dead after head trauma. This method has been used in the north of Lanzarote, as reported in interviews conducted by the authors with elderly residents of the area (personal communication). The multifocal incised wounds and subcutaneous hemorrhages observed in the animals from the first episode would be expected if ferrets were used during the poaching episode [31].

## 5. Conclusions

We present a forensic investigation of two different cases of illegal hunting of Cory’s shearwater in the Canary Islands. The results allow to conclude that the manner of death of this animal was non-natural; however, the study presents limitations due to the small sample size, decomposition of the carcasses, and the lack of controls and experimental confirmations.

## Figures and Tables

**Table 1:** Summary of individual weight, event, location data and relevant dates.

Case Nº	Island ^1^	Event	Sex ^2^	Weight (g)	Date Seized	Date of Reception
1	LNZ	1	M	1198	26 September 2020	8 October 2020
2	LNZ	1	M	856.4	26 September 2020	8 October 2020
3	LNZ	1	F	856.4	26 September 2020	8 October 2020
4	LNZ	1	M	1042.6	26 September 2020	8 October 2020
5	FTV	2	M	871.4	29 October 2020	18 November 2020
6	FTV	2	F	558	29 October 2020	18 November 2020
7	FTV	2	M	791	29 October 2020	18 November 2020
8	FTV	2	M	745	29 October 2020	18 November 2020
9	FTV	2	M	800	29 October 2020	18 November 2020
10	FTV	2	F	568.7	29 October 2020	18 November 2020
11	FTV	2	F	624.2	29 October 2020	18 November 2020
12	FTV	2	F	642.7	29 October 2020	18 November 2020
13	FTV	2	M	630	29 October 2020	18 November 2020
14	FTV	2	M	685.5	29 October 2020	18 November 2020
15	FTV	2	M	569.7	29 October 2020	18 November 2020
16	FTV	2	F	719.5	29 October 2020	18 November 2020
17	FTV	2	M	808	29 October 2020	18 November 2020
18	FTV	2	M	566.2	29 October 2020	18 November 2020
19	FTV	2	M	452	29 October 2020	18 November 2020
20	FTV	2	M	840	29 October 2020	18 November 2020

^1^ FTV: Fuerteventura; LNZ: Lanzarote; ^2^ M: male; F: female.

**Table 2:** Main pathological findings in Atlantic Cory’s shearwater (*Calonectris borealis*).

Finding	Episode 1 (*n* = 4)	Episode 2 (*n* = 16)	*p*-Value
**SKULL FRACTURE**	4	16	1.000
**CRANIAL HEMORRHAGE**	4	16	1.000
**BLOODSTAINED PLUMAGE**	4	15	1.000
**SKELETAL MUSCLE NECROSIS**	4	13	0.876
**ECTOPARASITES**	1	13	0.113
**RENAL TREMATODIASIS**	1	9	0.576
**BLOOD IN OROPHARYNX**	0	10	0.094
**CARDIAC HEMORRHAGE**	1	8	0.736
**MICROPLASTICS**	0	5	0.519
**FEATHERS IN ORAL CAVITY**	4	0	0.00016
**INCISED WOUNDS**	4	0	0.00016
**MYOCARDIAL DEGENERATION**	4	0	0.00016
**PULMONARY HEMORRHAGES**	0	3	0.876

## Data Availability

All data used in the current study are available from the corresponding author on reasonable request. The images of the necropsy and histopathology, and the head CT of the case 2 and case 6 are accessible at: https://zenodo.org/records/19372768 (accessed on 19 April 2026).

## Abbreviations

The following abbreviations are used in this manuscript:

IUCN	International Union for the Conservation of Nature
LESPRE	List of Wild Species under Special Protection Regime
CT	Computed tomography
FTV	Fuerteventura
LNZ	Lanzarote
